# Diffuse Large B-Cell Lymphoma Treated With R-CHOP in a Resource-Limited Setting in South Africa: A Real-World Study

**DOI:** 10.1093/oncolo/oyad069

**Published:** 2023-04-13

**Authors:** Zola Musimar, Mtonga Mpetani, Jeremy S Abramson, Bruce A Chabner, Zainab Mohamed

**Affiliations:** Radiation Oncology Department, Groote Schuur Hospital, University of Cape Town, Cape Town, Western Cape, Republic of South Africa; Radiation Oncology Department, Groote Schuur Hospital, University of Cape Town, Cape Town, Western Cape, Republic of South Africa; Massachusetts General Hospital Cancer Center and the POETIC Fellowship Exchange Program, Dana Faber, Harvard Cancer Center, Boston, MA, USA; Massachusetts General Hospital Cancer Center and the POETIC Fellowship Exchange Program, Dana Faber, Harvard Cancer Center, Boston, MA, USA; Radiation Oncology Department, Groote Schuur Hospital, University of Cape Town, Cape Town, Western Cape, Republic of South Africa

**Keywords:** lymphoma, rituximab, diffuse large B-cell lymphoma, global health, South Africa

## Abstract

**Background:**

Diffuse large B-cell lymphoma (DLBCL) is the most common subtype of non-Hodgkin’s lymphoma worldwide and particularly in Africa, where the incidence of HIV is the highest in the world. R-CHOP is the standard of care regimen for DLBCL, but access to rituximab is limited in developing countries.

**Methods:**

This is a retrospective cohort study that included all HIV-negative patients with DLBCL who received R-CHOP at a single institution from January 2012 to December 2017. Clinical and demographic data were collected to assess factors that influenced survival.

**Results:**

Seventy-three patients were included. Median age was 55 (17-76), 67.1% of patients were younger than 60 years, and 60.3% were female. Most presented with stages III/IV disease (53.5%) but with good performance status (56.% PS 0 and 1). Progression-free survival at 3 and 5 years was 75% and 69%, and overall survival at 3 and 5 years was 77% and 74%, respectively. Median survival had not been reached with a median follow-up of 3.5 years(0.13-7.9). Overall survival was significantly affected by performance status (*P* = .04), but not by IPI or age. Survival was significantly associated with response to chemotherapy after 4-5 cycles of R-CHOP (*P* = 0.005).

**Conclusions:**

Treatment of DLBCL with R-CHOP is feasible and can achieve good outcomes in resource-limited settings with rituximab-based chemotherapy. Poor performance status was the most important adverse prognostic factor in this cohort of HIV-negative patients.

## Teaser

R-CHOP is the standard of care regimen for diffuse large B-cell lymphoma (DLBCL), but access to rituximab is limited in developing countries. This article reports a study of patients with DLBCL, who received R-CHOP therapy and assesses factors that influenced survival.

**Implications for Practice**

Diffuse Large B-cell Lymphoma (DLBCL) is the most common subtype of Non-Hodgkin’s lymphoma (lymphatic system cancer) worldwide and particularly in Africa where the number of HIV patients is the highest in the world. R-CHOP is the most effective regimen for DLBCL, but access to rituximab a monoclonal antibody is limited in developing countries due to it cost. In this study we find that patients with poor ECOG performance status benefited less than adults while treated with R-CHOP, therefore performance status is the most important prognostic factor that could be used to guide the use of rituximab in resource-limited settings.

## Introduction

Diffuse large B-cell lymphoma (DLBCL) is characterized by the malignant proliferation of mature B-lymphocytes. It is the most common subtype of Non-Hodgkin’s lymphoma worldwide accounting for 30%-40% of all newly diagnosed cases.^1^ DLBCL is categorized according to morphology, immunohistochemical, and molecular characteristics.^2,3^ The relative incidence of DLBCL as compared to other subtypes of B-cell Non-Hodgkin’s lymphoma, in Africa is higher than in the developed world^4^ mainly due to the high incidence of HIV.^5^

Rituximab, cyclophosphamide, adriamycin, vincristine, and prednisone (R-CHOP) is the standard regimen for DLBCL.^1,6^ Rituximab, a chimeric monoclonal antibody against CD20 found on the surface of B lymphocytes,^7^ was initially added to the CHOP regimen for the treatment of patients with DLBCL in the early 2000s and improved overall and progression-free survival in randomized controlled trials.^8,9^ In high-income countries, the addition of rituximab to CHOP conferred an absolute benefit of 10%-20% in complete response and survival when treating patients with DLBCL.^10^ This finding has not been confirmed in low- and middle-income countries (LMIC) in Africa,^11^ where resource constraints limit oncology care and access to expensive drugs^12^ Optimal quality of care which must include strong support services and compliance with protocol therapy, is essential to ensuring satisfactory treatment outcomes particularly in patients who have a poor performance status (PS) and in elderly patients who may have multiple co-morbidities.^13^ Patient compliance with complex regimens, access to drugs and clinics, and other aspects of optimal quality of care are often lacking in resource-constrained settings^14^ and can undermine the effectiveness of internationally accepted standard-of-care drug therapies.^15^ Therefore, this retrospective study in South Africa (SA), an upper-middle-income country with an under-resourced state health sector, was conducted to assess whether standard combination chemoimmunotherapy results in similar outcomes to high-income countries.

“Real-world” studies looking at the cost-effectiveness of rituximab have been conducted in other resource-limited countries.^16,17^ A study from Mexico, classified by the World Bank as an upper-middle-income country like South Africa (World bank 2021), demonstrated no significant benefit in terms of overall survival for the addition of rituximab in DLBCL.^18^ Therefore, this study aimed to evaluate the outcomes of R-CHOP treatment in a public health institution in an African middle-income country with multiple competing health priorities.^19^

We also hoped to determine whether the International Prognostic Index (IPI), which was developed as a prognostic tool for the management of aggressive B-cell lymphomas in the pre-rituximab era,^20^ could be useful as a guide to prognosis in our patient care setting. Poor prognostic IPI factors are each scored 1 and include patient age >60 years, elevated lactate dehydrogenase (LDH), ECOG performance status ≥2, Ann Arbor stages 3 and 4, and the presence of >1 extranodal disease site. The MINT and GELA trials demonstrate a 10%-15% improvement in complete remission and overall survival rate at 2 years with R-CHOP as compared to CHOP regardless of the International Prognostic Index (IPI) at diagnosis.^21^ However, a newer version of the IPI that incorporated specific extra-nodal sites (bone marrow, liver, etc.), defined age groups, and LDH levels is now widely used, and a multicenter analysis concluded that the NCCN IPI is currently the best prognostic tool for DLBCL.^22^

In low- and middle-income countries rituximab is not always available and frequently not optimally used in the public sector due to cost and problems in equitable healthcare delivery.^23^ R-CHOP has proved to be superior to CHOP alone in health economic studies,^24^ primarily because the cost of retreatment after relapse and the impact of life years lost outweighs the cost of its incorporation into first-line curative treatment.^16^ Limitation in the use of rituximab in the public sector of health care holds true in SA where rituximab is not available for all standard indications due to the cost of this drug. At our institution, due to budget limitations during the study period, rituximab was only being used for HIV-negative patients and was preferably given to younger patients with good PS and no other major comorbidities. From this analysis, we hoped to find a prognostic factor for those who would derive substantial benefit from the addition of rituximab.

## Materials and Methods

This was a retrospective cohort study based on “real world data” and including all consecutive HIV-negative patients with previously untreated DLBCL who received R-CHOP at the Department of Radiation Oncology, Groote Schuur Hospital in Cape Town, SA from January 2012 to December 2017.

Our objectives were to define the demographic and clinical characteristics of DLBCL patients receiving R-CHOP, assess their overall survival, complete response rate, and progression-free survival, and determine the impact of IPI, performance status, and age on overall survival

All the patients included in this study we diagnosed based on an incisional or excisional biopsy.

Fine needle aspiration was not accepted as a lymphoma diagnostic procedure. Biopsy samples were subjected to immunohistochemistry (CD20, CD10, CD3, Bcl2, Bcl6, and Ki67). For some biopsies, CD45, CD23, CD30, CD5, cyclin D1, EBER, TDT, and other histochemical tests were used, to differentiate lymphoma subtypes. CD20 positivity was required for patients receiving rituximab. Staging was performed utilizing CT or PET CT imaging and bone marrow aspirate and trephine biopsy. The standard IPI was calculated for all patients with available data.^25^

Subjects were identified using the Department of Radiation Oncology Electronic Patient Registry (EPR). Institutional Human Research Ethics committee approval was granted before proceeding with this study.

Overall survival was defined as the time from initial presentation (date of biopsy) to the date of the last encounter or death. Those who were lost to follow-up were censored at the date last seen. In this institution, response to chemotherapy was assessed with a CT scan or 18-FDG-PET-CT performed after completing 4-5 cycles of R-CHOP.

Relapse-free survival was defined as the time from the last date of chemotherapy to the date of relapse

Progression-free survival was defined here as the length of time from the date of diagnosis to the time of disease progression or death regardless of cause.

For this study, Cheson response criteria were used for patients staged based on clinical evaluation and a CT scan. Complete response was defined as the absence of all evidence of disease by physical examination and imaging at the time of interim evaluation (4-5 cycles therapy).^26^ When using PET CT for interim imaging the Lugano criteria were used; a complete response was defined as a Deauville score of 1, 2, or 3 with or without a residual mass and no evidence of FDG-avid disease in the bone marrow.^27^ Complete response rate was defined as the percentage of patients with a complete response at the time of the interim scan after 4-5 cycles of therapy. In our institution, those with complete response were planned to complete 6 cycles of chemotherapy. Patients with no or partial response (Deauville 5) after 4-5 cycles of chemotherapy were considered for salvage therapy. Those younger than 65 years with well-controlled comorbidities and adequate organ function were offered high-dose salvage chemotherapy with a view to stem cell transplant. Patients with partial response (Deauville 4) at interim analysis completed the planned 6 cycles of chemotherapy and were evaluated at that point

For partial responders after 4-5 cycles of therapy, we were unable to document the complete response rate at end of 6 cycles of treatment as we do not routinely repeat imaging at that point. It is important to note that the follow-up ceased in March 2020 due to the COVID-19 pandemic.

### Data Collection

Clinical and demographic data were collected from patient files as well as the National Health Laboratory Services and Radiology databases. Survival data were obtained from patient files and the Groote Schuur Hospital Clinicom system. Data collected included age, sex, initial PS (ECOG), Ann Arbor stage, IPI, number of cycles of chemotherapy, response to treatment, date of relapse, and death. Comorbidities were defined as any chronic medical condition that could impact the ability to receive treatment, eg, hypertension, diabetes, ischemic heart disease, tuberculosis, etc. Laboratory data collected at baseline included hemoglobin, white cell count, absolute neutrophil count, platelet count, serum albumin and lactate dehydrogenase, liver and kidney function tests, and routine chemistries.

### Statistical Analysis

Descriptive statistics were performed to analyze demographic and clinical data (age, sex, stage, performance status, and IPI). Kaplan-Meier survival analysis was performed to assess overall, progression-free, and relapse-free survival. The log-rank test was used as a test of equality of survival distributions for the different levels of IPI, performance status, and age. Lastly, a chi-square test for association was used to determine whether there was an association between survival and sex, response by 4-5 cycles, co-morbidities, toxicity, histological subtype, and treatment. (Fischer’s Exact test was interpreted if the chi-square requirements were not met). A Bonferroni correction was made due to multiple comparisons with the same dependent variable, this correction decreases the possibility of making a type I error.

## Results

**Figure 1.**
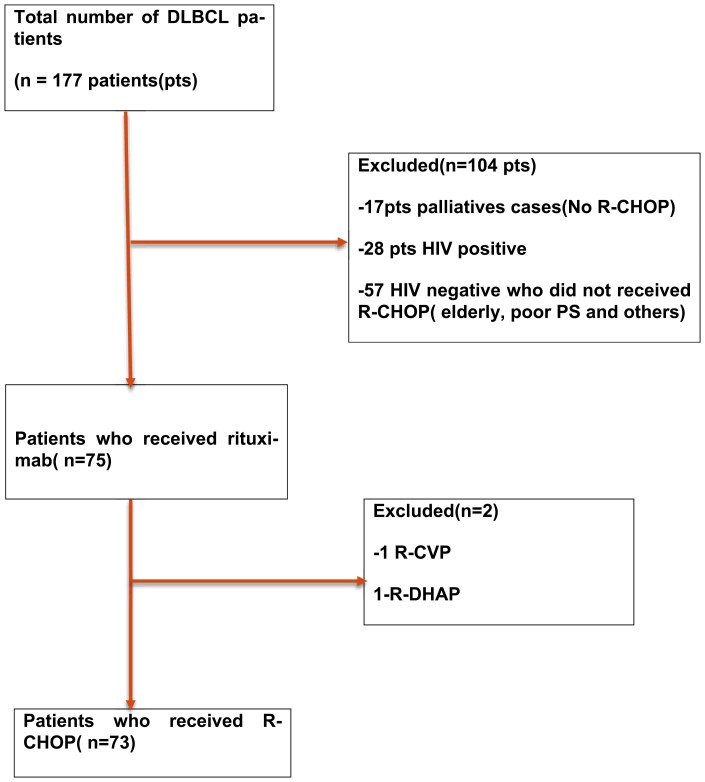
CONSORT diagram: flow diagram of patients data collection.

A total of 177 patients diagnosed with DLBCL from January 2012 to December 2017 were identified (Fig. 1), of which 73 patients who received R-CHOP met the inclusion criteria, for this analysis. One hundred and four patients were excluded from the analysis for the following reasons: HIV negative who did not receive R-CHOP (poor PS, co-morbidities, or elderly) (*n* = 57); HIV positive (*n* = 28), or chose palliative treatment (*n* = 17). Two patients received rituximab but not with CHOP (R-DHAP and R-CVP)

### Patients Characteristics

The median age was 55 years (range 17-76) with most patients (*n* = 48, 67.1%) being 60 years or younger. The cohort was predominantly female (58.9%). The majority presented with an advanced Ann Arbor stage, specifically 28.8% with stage III and 24.7% with stage IV disease. Stage II was the commonest stage at presentation (38.4%).

Most patients had a good performance status, 56% being ECOG 0-1 (Table 1).

**Table 1.** Baseline characteristics.

Characteristic	Frequency (*n*)	Percent/range
Age, years (*n* = 72)		
Median	55	(17-76)
Sex (*n* = 72)		
Male	29	39.7
Female	44	60.3
Total	73	100
Stage (*n* = 73)		
I	6	8.2
II	28	38.4
III	21	28.8
IV	18	24.7
Total	73	100
ECOG performance status		
0	9	12.3
1	32	43.8
2	20	27.4
3	8	11.0
4	3	4.1
Total	72	98.6
Missing	1	1.4
	73	100
IPI		
0	4	5.5
1	20	27.4
2	22	30.1
3	17	23.3
4	4	5.5
Missing	6	8.2
Total	73	100

### Treatment Outcomes

Interim response after cycles 4 or 5 was assessed in 72 out of 73 patients. Complete response after 4-5 cycles of R-CHOP was reported in 34 patients (47.2%) and a partial response in 38 patients (52.8%).

The last scan for most patients was after 4-5 cycles of chemotherapy (interim scan); therefore, we could have underestimated the complete response rate, as most patients had full 6 cycles of therapy.

**Figure 2.**
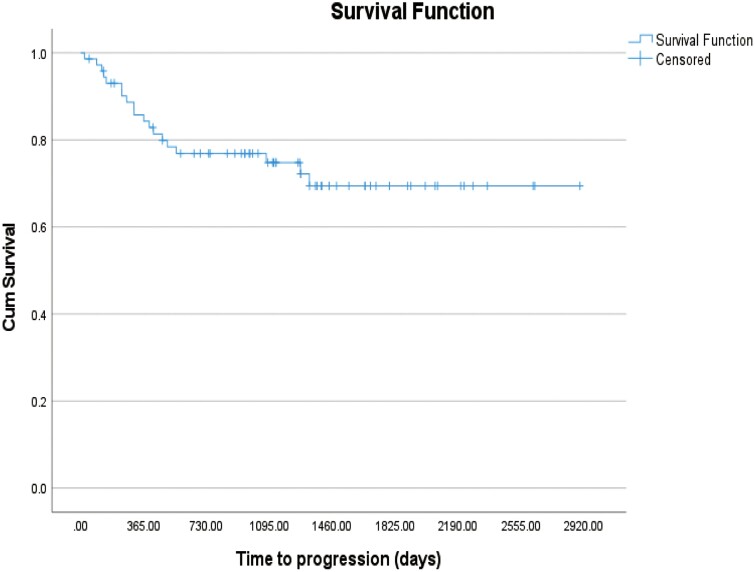
Progression-free survival.

The median follow-up time was 3.5 years (range 0.13-7.9). Estimated progression-free survival at 3 and 5 years was 75% and 69%, respectively (Fig. 2). A total of 17 patients (23.3%) had died at the time of this analysis. The estimated overall survival from the date of diagnosis was 77% at 3 years and 74% at 5 years. Death was lymphoma-related in 14 patients, treatment-related in 2, and due to second malignancy in 1 patient. Median survival time has not been reached.

Radiotherapy was used to treat 27 patients (37%). Involved site radiotherapy with curative intent was used in 21 patients either for consolidation in early stage, previously bulky, or localized residual disease. It was used as palliation in 6 patients. In the curative intent radiotherapy, group 3 of 21 patients died after relapse; and in the palliative group 2 out of the 6 were still alive at the end of the study period. Two relapsed patients received high-dose chemotherapy, anticipating autologous stem cell transplant, but both experienced disease progression and did not receive stem cell transplant.

Relapse was reported in 11 out of 73 patients (15.1%) including 4 of 34 patients with a complete response and 7 of 38 patients with a partial response after initial R-CHOP.

The cumulative survival for patients with progressive disease was 27% at 2 years after diagnosis was 27% and was 18% at 5 years. For those who are in a continuous complete or partial response survival was 85% at 5 years after the date of diagnostic.

**Figure 3.**
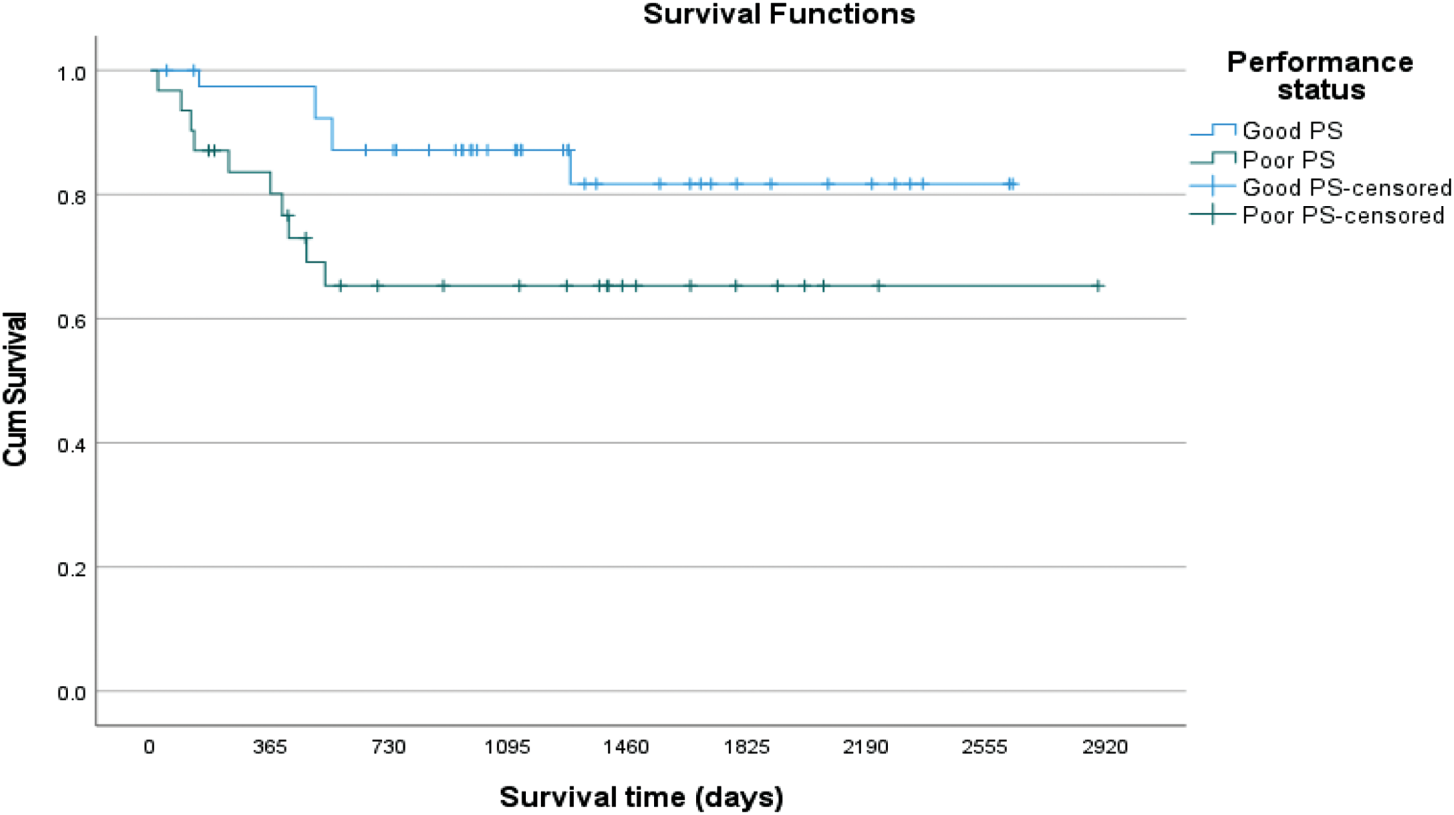
Kaplan-Meier survival curve showing the impact of PS on survival.

**Figure 4.**
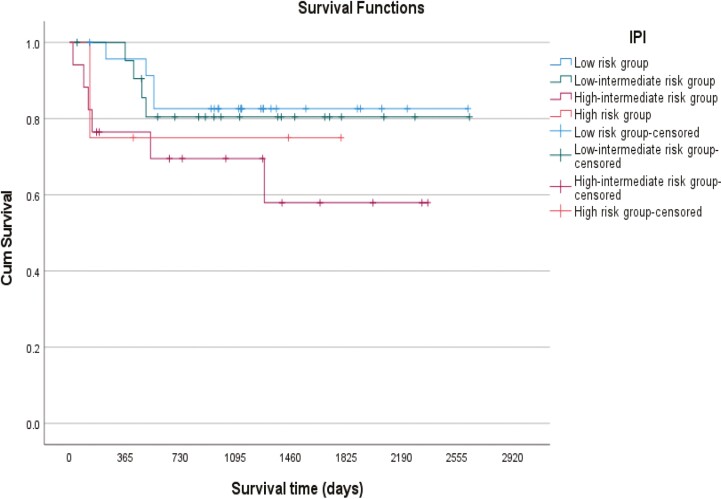
Kaplan-Meier survival curve showing the impact of IPI on survival.

**Figure 5.**
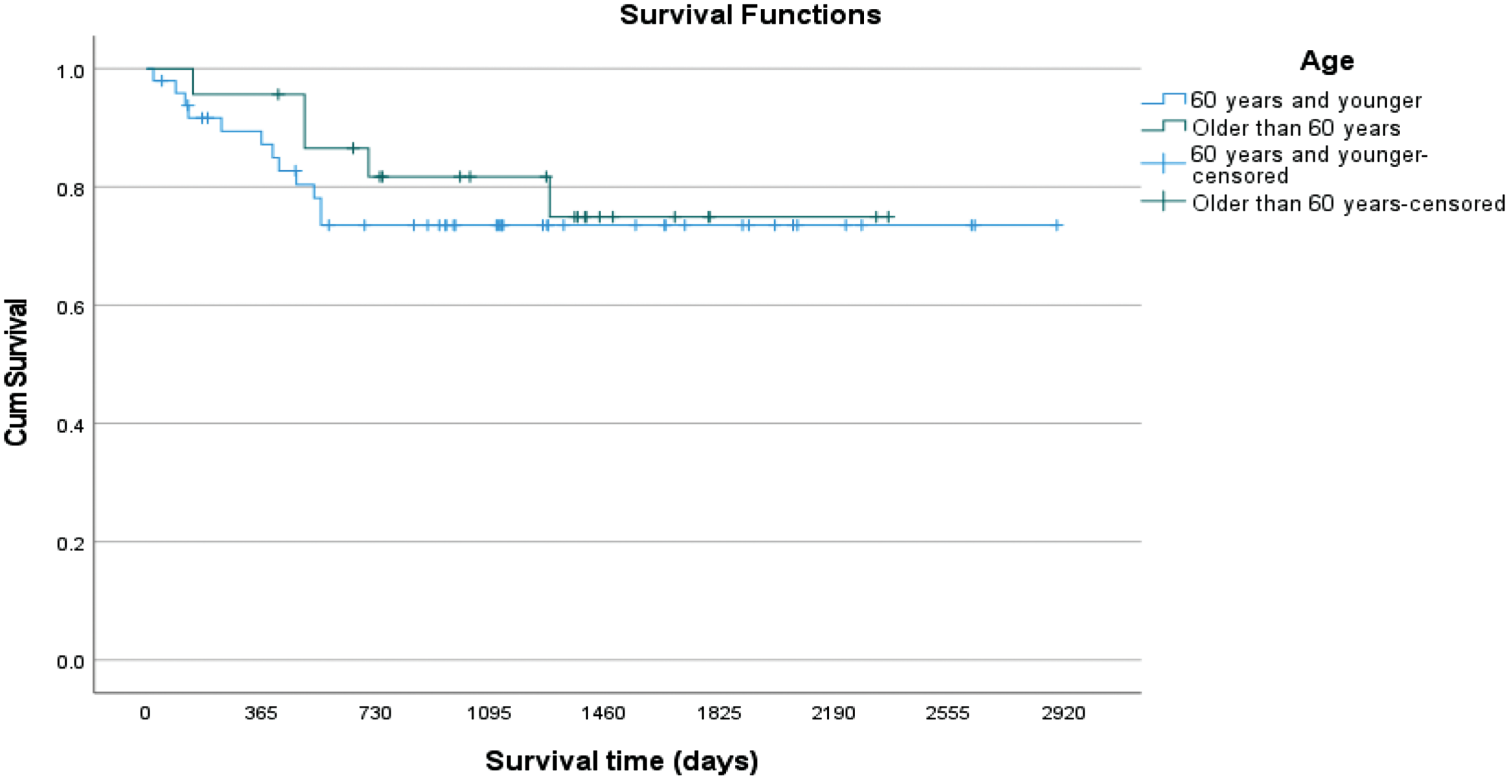
Kaplan-Meier survival curve showing the impact of Age on survival.

The impact on survival of IPI, performance status (PS), and age at the time of diagnosis was analyzed. The survival distributions for patients with PS ECOG 0-1 were statistically better than for those with a PS of 2-4 (log rank *P* = 0.038) but neither IPI (log rank *P* = 0.359) nor age (log rank *P* = 0.576) affected survival in this analysis. The survival at 2 and 5 years after the date of diagnosis for patients with a good performance status (ECOG = 0-1) was 87% and 82%, respectively, while for patients with a poor performance status (ECOG = 2-4) had an overall survival of 65% at 2 years that was maintained at 5 years after the date of diagnosis (Figs. 3, 4, and 5).

There was a statistically significant association between survival and response after 4-5 cycles (Chi-square *P* = 0.005). In contrast to 63.2% of patients who had an incomplete response at this stage, 91.2% of patients who had a complete response at this point were still living after 2 years (*P* = .005).

## Discussion

Real-world data from low- and middle-income countries on patients with DLBCL treated with international standard-of-care therapies are lacking.^28^ This retrospective study is a long-term follow-up of patients with DLBCL treated at a single institution over a 5-year period (2012-2017) after rituximab became available for public sector use in South Africa. At the time of the study, HIV-positive patients did not receive rituximab due to cost constraints and lack of clarity on whether rituximab offered benefits in this patient population.

Most patients included in this study (67.1%) were younger than 60 years with a median age of 55 years. In the developed world patients with DLBCL tend to be older than 60 as demonstrated in a study conducted in the United Kingdom where the median age of patients with newly diagnosed DLBCL was 70.2 years.^29^ Although some patients were excluded from treatment on the basis of their age in this study, the younger age at presentation among Southern Africans with DLBCL has been noted by other researchers both during^30^ and prior^31^ to the HIV epidemic. Socioeconomic challenges affecting health-seeking behavior and access to cancer care as well as unexplored biological features could explain this finding. Our institution services the uninsured population who mostly come from disadvantaged communities with limited life expectancies.^32,33^ In addition, South Africa has a young population with an average life expectancy of 64 years in 2019 according to the Statistics South Africa midyear population estimate.^34^

The projected 5-year overall survival of 74% and progression-free survival of 69% compare favorably to that achieved at better-resourced institutions outside of Africa.^1,7^ In contrast 5-year OS in HIV-negative patients with DLBCL in a study conducted at this institution prior to the introduction of rituximab was 56%.^35^ While the survival estimates in the current study are based upon a relatively small retrospective cohort of patients with a median follow-up of 3.5 years, this result suggests that standard treatment including rituximab may improve survival in spite of the limited healthcare resources in South Africa.^29^

Radiotherapy was used in a third of the cohort and may have positively affected survival outcomes. Most recently PET-directed radiotherapy has yielded excellent results,^36^ when a part of treatment regimens for patients with limited stage non-bulky DLBCL when used with 2-4 cycles of R-CHOP. Radiation therapy may still be considered for sites of refractory disease, as for example for consolidation for limited-stage bulky disease, or as a palliative tool for relapsed disease. Radiotherapy is a valuable resource in LMICs for consolidative or palliative treatment of DLBCL in settings where stem cell transplant, CAR-T cell, and other novel therapies are not readily available.

Survival for relapsed patients was 18% at 5 years which is worse than that found by a Swedish group and others.^37^ Other literature has suggested that poor compliance and treatment interruptions in low-resource settings (Africa) are associated with recurrence or disease progression and in turn survival.^38^ These data also reflect the need for improved treatments of DLBCL in the relapsed/refractory setting, including high-dose chemotherapy with bone marrow replacement and emerging therapies such as antibody-drug conjugates and CAR T-cells.

We evaluated the impact of IPI in general, as well as age and PS, on survival to see if the commonly accepted prognostic factors hold true in our setting. Survival was significantly associated with performance status but not with IPI or age in this selected population of DLBCL patients. Lack of a statistically significant effect of IPI on survival may reflect the small sample size rather than the true lack of impact of IPI on survival outcome, as there is a trend of inferior survival in high-risk IPI patients. However, similar findings have been described in other studies where PS was more important in predicting outcomes than age.^29,39^ Similarly, IPI as shown by the GELA trial, was not a significant factor in predicting the overall survival rate at 2 years for patients treated with R-CHOP,^21,40^ a finding which led to the development of newer prognostic tools like the NCCN IPI.^22^

Patients who present with ECOG PS 2-4 experienced poor survival despite treatment with R-CHOP. Therefore, a rational approach to prescribing rituximab should be considered in low and middle-income settings where cost-benefit ratio is crucial. Older patients with ECOG PS 0-1 responded as well to R-CHOP as those younger than 60 years so advanced age should not be a criterion for withholding rituximab. Real-world studies conducted in Asia^39,41^ found a poorer outcome for patients older than 70-75 years, while, studies conducted in Denmark^42^ and Sweden^43^ showed good outcomes in the over 75-year age group. R-miniCHOP could be considered in the over 80 age group.^44^ The addition of consolidative radiotherapy may also demonstrate benefit in patients over 60.^45^

A complete response after 4-5 cycles of R-CHOP was significantly associated with longer survival on univariate analysis. PET CT was used for staging and interim assessment in approximately 80% of our cohort. It is an excellent tool for lymphoma staging and outperforms CT in response assessment,^46^ but, is not routinely available in most African countries.^47^ There is still some debate about the prognostic value of PET CT for DLBCL.^48,49^ The UK National Cancer Research Institute found an early complete metabolic response to be associated with complete response at end of treatment, but patients with a Deauville score of 5 after 2 cycles of R-CHOP were the only groupg in which survival was adversely affected.^50^ The S1001 study suggested the advantage of using complete response on PET in early-stage, non-bulky DLBCL patients as a criterion to limit treatment to 4 cycles of R-CHOP without the addition of radiotherapy.^36^

## Conclusions

This real-world study in a middle-income country illustrates the younger demographic affected by DLBCL in South Africa and suggests favorable outcomes for R-CHOP in DLBCL. We find that ECOG performance status is the most important prognostic factor that could be used to guide the use of rituximab in resource-limited settings. Given the inherent weaknesses of retrospective analyses, we advocate for the conduct of prospective clinical trials of cancer therapy in low- and middle-income countries.

## Data Availability

The data underlying this article will be shared on reasonable request to the corresponding author.
